# Dairy-Rich Diets Augment Fat Loss on an Energy-Restricted Diet: A Multicenter Trial

**DOI:** 10.3390/nu1010083

**Published:** 2009-09-03

**Authors:** Michael B. Zemel, Dorothy Teegarden, Marta Van Loan, Dale A. Schoeller, Velimir Matkovic, Roseann M. Lyle, Bruce A. Craig

**Affiliations:** 1 The University of Tennessee, Knoxville, TN 37996, USA; 2 Purdue University, 700 W. State St., W. Lafayette, IN 47907, USA; Email: dteegard@purdue.edu (D.T.); rlyle@purdue.edu (R.M.L.); bacraig@stat.purdue.edu (B.A.C.); 3 USDA, ARS, Western Human Nutrition Research Center, 430 W. Health Sciences Drive, Davis, CA 95616, USA; Email: Marta.VanLoan@ars.usda.gov; 4 The University of Wisconsin, 1415 Linden Drive, Madison, WI 53706, USA; Email: dschoell@nutrisci.wisc.edu; 5 Ohio State University, 480 W. 9^th^ Ave, Columbus, OH 43210, USA; Email: matkovic.1@osu.edu

**Keywords:** calcium, dairy, energy restriction, multi-center, vitamin D

## Abstract

A 12-week randomized controlled multi-center clinical trial was conducted in 106 overweight and obese adults. Diets were designed to produce a 2,093 kJ/day energy deficit with either low calcium (LC; ~600 mg/day), high calcium (HC; ~1,400 mg/day), or high dairy (HD; three dairy servings, diet totaling ~1,400 mg/day). Ninety-three subjects completed the trial, and 68 met all *a priori* weekly compliance criteria. Both HC and HD contained comparable levels of calcium, but HC was only ~30% as effective as HD in suppressing 1,25-(OH)_2_D and exerted no significant effects on weight loss or body composition compared to LC. In the group that met compliance criteria, HD resulted in ~two-fold augmentation of fat loss compared to LC and HC (HD: -4.43 ± 0.53 kg; LC: -2.69 ± 0.0.53 kg; HC: -2.23 ± 0.73kg, p < 0.025); assessment of all completers and an intent-to-treat analysis produced similar trends. HD augmentated central (trunk) fat loss (HD: -2.38 ± 0.30 kg; HC: -1.42 ± 0.30 kg; LC: -1.36 ± 0.42 kg, p < 0.05) and waist circumference (HD: -7.65 ± 0.75 cm; LC: -4.92 ± 0.74 cm; LC: -4.95 ± 1.05 cm, p < 0.025). Similar effects were noted among all subjects completing the study and in an intent-to-treat analysis. These data indicate that dairy-rich diets augment weight loss by targeting the fat compartment during energy restriction.

## 1. Introduction

Data from several studies suggest that dietary calcium and dairy foods may modulate energy partitioning and adipocyte lipid metabolism, resulting in reductions in body weight and fat [1]. We first reported that increasing dietary calcium significantly augmented weight and fat loss secondary to a fixed level of energy restriction (a 2,093 kJ/day deficit) [2]; increasing dietary calcium intake from ~400 to ~1,200 mg/day resulted in 26 and 28% increases in weight and fat loss, respectively, over a 24-week period, while utilizing dairy foods in place of calcium supplements produced significantly greater effects, with weight and fat loss augmented by 64 and 70% [2]. Similarly, a shorter-term (12-week) follow-up trial demonstrated that incorporation of sufficient yogurt into the daily diet to raise dietary calcium from ~500 mg/day to ~1,100 mg/day without changing dietary macronutrients augmented fat loss during energy restriction by 61% while preserving lean mass [3]. These findings were also replicated in a six-month clinical trial in obese African Americans [4] in which inclusion of three daily servings of dairy into an energy-restricted diet resulted in ~two-fold increases in weight, fat and trunk fat loss versus those maintained on a low dairy diet. In contrast, a retrospective analysis of three calcium supplementation trials originally conducted to investigate the effects of calcium on skeletal outcomes during energy restriction reported no significant effect on weight loss or body fat mass [5]. However, the authors noted that the magnitude and direction of the observed changes in body weight and fat were consistent with a hypothesized effect that would be detectable with a larger sample size [5]. In addition, in a 48-week randomized controlled weight loss trial, increasing the dairy product intake of subjects consuming a moderate level of calcium (~800 mg/day) and dairy during energy restriction did not alter weight loss; however, a low calcium/low dairy comparison group was not included [6]. 

These effects have also been examined in the absence of calorie restriction. Isocaloric substitution of three daily servings of dairy products into the diets of obese African-Americans for six months resulted in a 5.4% reduction in total body fat and a 4.6% decrease in trunk fat (p < 0.01 for both) in the absence of any change in body weight while the control group maintained on a low calcium/low dairy diet with identical macronutrient composition exhibited no significant changes in total body fat or trunk fat [4]. In contrast, a 12 month randomized controlled trial demonstrated no effect of isocaloric substitution of dairy products into diets of normal weight young women with low calcium intakes [7]. 

Although only limited data are available from prospective, randomized trials, additional evidence from retrospective clinical observations, epidemiological studies, rodent studies and mechanistic studies further suggest an anti-obesity effect of dietary calcium and dairy foods [1]. Although some of the randomized controlled clinical trials originally designed to evaluate the effects of dietary calcium on skeletal endpoints do not support an effect of calcium supplementation on body weight [8], several studies in younger and older women [9,10] and in obese men [11] provide findings which suggest an effect of calcium or dairy products on body weight. Similarly, several prospective studies, including a two-year study of normal weight women [12], ten-year data from the CARDIA study [13], and an eight-year study of children studied from two months of age [14,15] support these findings, as do epidemiological observations from the Quebec Family Study [16], the Continuing Survey of Food Intake by Individuals [16], NHANES 1999-2000 [17] and the Heritage Family Study [18]. Moreover, controlled rodent studies demonstrate that inclusion of increased calcium or calcium-rich dairy products into mouse diets attenuated diet-induced obesity [11], accelerated both weight and fat loss secondary to caloric restriction [19], and reduced weight and fat regain during ad libitum re-feeding following weight loss [20], with dairy sources of calcium exerting greater effects.

Although multiple lines of evidence support an effect of dairy products or calcium supplementation on body weight and composition, there have been only a small number of randomized clinical trials, and the results of these trials are not uniform. Accordingly, the present study was designed as a larger-scale, multi-center study to determine the roles of supplemental calcium and dairy foods in augmenting fat and weight loss secondary to energy restriction in overweight and obese adults.

## 2. Materials and Methods

*Study Design:* This study was designed to determine whether dairy products or calcium would accelerate weight and fat loss induced by caloric restriction in 106 otherwise healthy overweight and obese young adults. Subjects were studied for a two-week lead-in period to establish their current caloric requirements and provide an opportunity for baseline dietary and physiological assessment, and then randomized to the following outpatient dietary regimens for 12 weeks: (1) a control diet providing a 2,093 kJ/day deficit, 0-1 servings of dairy products/day, 500 mg calcium per day, and a daily placebo (methyl-cellulose) supplement; (2) a calcium-supplemented diet identical to the control diet, with the placebo replaced by 900 mg calcium in the form of calcium carbonate to increase total calcium intake from 500 to 1,400 mg/day; or (3) a high dairy diet (placebo supplemented) providing a 2,093 kJ/day deficit and containing three daily servings dairy products (milk, cheese and/or yogurt) substituted for other protein sources in the diet, to bring the total calcium intake from 500 to 1,400 mg/day without altering macronutrient intake. The first two arms of the study were conducted in a placebo-controlled, blinded fashion, while the third arm (dairy) was by necessity unblinded. However, subjects on the high dairy diet also received a placebo supplement and all groups were treated as active-treatment arms, with pill counts serving as a component of the compliance measurement, as indicated below. Supplements (calcium or placebo) were provided as three daily doses taken with meals.

Physical activity and tobacco use were assessed using standard questionnaires and maintained at pre-study (baseline) levels throughout the study. Body weight, waist circumference, blood pressure and heart rate were measured weekly, with subjects wearing street clothes with no shoes, outerwear or accessories. Body weight measurements at baseline and week 12 were done in conjunction with body composition measurements, with subjects wearing surgical scrubs. Body fat was measured at the beginning of the study and at week 12 using dual x-ray absorptiometry (DXA); DXA was also utilized to ascertain regional fat loss (trunk fat versus other regions). Fasting levels of circulating insulin, glucose, glucose:insulin ratio (as a screening index of insulin sensitivity), calcium regulatory hormones (25-OH-D, 1,25-(OH)_2_-D, parathyroid hormone and calcitonin), blood pressure and fasting plasma lipids (triglycerides, total and HDL cholesterol) were measured at the same time points (baseline and week 12). 

*Subjects*: One hundred and six overweight and mildly obese individuals were recruited from the faculty, staff and student populations of each of the four participating institutions (University of Tennessee, Purdue University, The Ohio State University and the USDA, ARS, Western Human Nutrition Research Center at the University of California-Davis). Distribution of subjects across the four sites is shown in Table 1. 

**Table 1:** Distribution of Subjects.

	Purdue			Tennessee			USDA Davis			Ohio State			Total		
	LC^1^	HC^2^	HD^3^	LC	HC	HD	LC	HC	HD	LC	HC	HD	LC	HC	HD
**Enrolled**	11	11	12	9	9	9	9	9	9	9	7	2	38	36	32
**Completed**	11	9	11	9	7	8	8	7	9	8	6	0	36	29	28
**Adherent**	9	8	9	7	7	8	7	2	6	3	2	0	26	19	23

^1^ LC, Low Calcium ^2^ HC, High Calcium ^3^ HD, High Dairy

Inclusion criteria included 18-35 years of age, an initial body mass index (BMI) of 25-34.9 kg/m^2^; a low calcium diet at enrollment (<600 mg calcium/day from non-calcium fortified foods and <800 mg total calcium/day; no more than 3 kg weight change during past three months; and no recent (four weeks) changes in exercise intensity or frequency. Subjects were excluded from participation if they required the use of oral antidiabetic agents or insulin; used obesity pharmacotherapeutic agents and/or herbal or other preparations intended for use in obesity or weight management within the previous 12 weeks; used calcium supplements within the previous 12 weeks; had a history of significant endocrine, hepatic or renal disease; were pregnant or lactating; had a recent (past 12 weeks) initiation of or change in oral contraceptive or hormone replacement regimen; suffered any active form of malabsorption syndrome or had a history of eating disorders. Table 2 shows the baseline characteristics of enrolled subjects. Subjects were predominantly female (79%) and Caucasian (80%).

**Table 2:** Baseline Characteristics of Enrolled Subjects ^1^.

Parameters	LC	HC	HD	p
**Age (years)**	25.35 ± 4.88	26.24+4.82	25.55+4.99	0.7228
**Height (cm)**	165.53 ± 7.89	166.27+10.78	167.24+6.21	0.7072
**Gender**	30 Female/8 Male	30 Female/6 Male	24 Female/8 Male	0.7242
**Weight (kg)**	80.10 ± 12.40	82.74 ± 14.81	80.11 ± 12.16	0.6224
**BMI (wt/m^2^)**	29.35 ± 2.76	29.86 ± 2.57	28.78 ± 2.85	0.2748
**Fat Mass (kg)**	32.11 ± 4.73	32.38 ± 6.98	32.28 ± 7.35	0.9830
**Lean Mass (kg)**	44.54 ± 10.28	46.83 ± 11.53	44.53 ± 9.05	0.5627
**Waist Circum (cm)**	88.80 ± 10.67	90.37 ± 9.97	89.77 ± 10.34	0.8055
**25OHD (ng/mL)**	23.13 ± 4.79	21.84 ± 5.05	22.69 ± 5.07	0.6017
**PTH (ng/mL)**	21.34 ± 11.77	30.28 ± 15.87	27.16 ± 18.70	0.1229
**1,25(OH)2D (pg/mL)**	52.55 ± 4.76	50.81 ± 4.97	54.23 ± 4.05	*0.0240
**Dietary Ca (mg/day)**	496 ± 81.9	451 ± 105	488 ± 104	0.7244
**Glycerol (µMol/L)**	101.03 ± 32.96	77.55 ± 28.43	91.27 ± 30.08	*0.0148
**Diastolic BP (mm Hg)**	73.21 ± 6.41	70.6 ± 7.49	73.31 ± 8.00	0.2184
**Systolic BP (mm Hg)**	115.20 ± 11.09	110.86 ± 10.02	113.39 ± 10.45	0.2218
**Insulin (μU/mL)**	8.60 ± 3.39	10.13 ± 5.29	11.37 ± 6.61	0.1286
**Glucose (mM/L)**	4.75 ± 0.54	4.67 ± 0.42	4.78 ± 0.56	0.6825

^1^ mean ± standard deviation

This research was approved from an ethical standpoint by the Institutional Review Boards of each of the four participating institutions. Standardized informed consent was obtained from all subjects, and the research was conducted in accordance with the ethical standards outlined in the Helsinki Declaration.

*Diets*: Baseline dietary assessments (diet records) were conducted by the project dietitian at each site during the two-week lead-in period and were used to provide an initial estimate of a maintenance level of energy intake. Subjects were trained to complete diet records. The estimate of energy intake was then refined by calculating needs using World Health Organization equations for calculation of basal metabolic rate, adjusted for activity level to provide an estimate of total daily energy expenditure (TDEE). TDEE was calculated as 1.3 X BMR for subjects engaged in mild daily activity and 1.5 X BMR for those engaged in strenuous daily activity. Discrepancies between estimated TDEE and baseline caloric intake were resolved, if necessary, by repeat diet records reviewed by the project dietitians. Based on this initial estimate of caloric needs, a food exchange-based diet was prescribed to produce a caloric deficit of approximately 2,093 kJ/day. The diets for the treatment arms were constructed to provide comparable levels of macronutrient and fiber, to approximate the average consumption in the U.S. (fat, ~35% of total energy, carbohydrates ~49%, ~protein 16%, fiber 2-3 g/1,000 kJ/day). Nutritional supplements were not permitted, and caffeine intake was maintained at a constant level (individualized for each patient, based on baseline assessment). Diets were prescribed and monitored as noted above. Subjects in the high dairy group were permitted to utilize both full-fat and low-fat milk, cheese and yogurt, with the fat accounted for in exchange lists given with each individual diet prescription. 

Subjects were provided individual instruction, counseling and assessment from the study dietitian regarding dietary adherence and the development and reinforcement of strategies for continued success; although the diets were individualized to achieve a 2,093 kJ/person/day deficit, comparable advice was to be given to patients in all treatment groups and diets were monitored weekly. All subjects maintained complete diet diaries, and compliance was assessed by weekly subject interview and review of the diet diary and pill-counts. Energy and macronutrient intake during the study is summarized in Table 3; there were no significant differences among treatments, although mean energy and protein intake was somewhat higher in the HD group.

**Table 3:** Self-reported energy and macronutrient intake during intervention ^1^.

Parameter	LC	HC	HD
**Energy (kJ/d)**	5610 ± 1013	5895 ± 1239	6356 ± 1118
**Carbohydrate (g/d)**	178** ± **38	186 ± 46	191 ± 28
**Protein (g/d)**	60 ± 25	60 ± 13	71 ± 13
**Fat (g/d)**	42 ± 11	45 ± 10	49 ± 14

^1^ mean ± standard deviation

*Assessment*: Body weight was measured weekly with a calibrated scale and height measured with a wall-mounted stadiometer with subjects in street clothes with no outerwear or shoes. Weight measurements taken at baseline and week 12 were made in conjunction with the body composition measurements, described below, with subjects wearing surgical scrubs. Body mass index was calculated via standard equation (kg/m^2^). Waist circumference was measured in the standing position, with measurements obtained midway between the lateral lower rib margin and the iliac crest. The measurements were taken mid-exhalation, and the average of two readings was recorded. 

Total fat and lean mass were assessed via dual energy x-ray absorptiometry (DXA) at baseline and week 12 of the study. To ensure uniformity, each study site utilized identical equipment (Lunar Prodigy DXA, GE Lunar, Madison, WI), and all systems were installed and calibrated by the same Lunar staff in Spring, 2001. The study staff at each center was trained and certified in DXA assessment of both body composition and bone density. At each site, a spine phantom was assessed every day to determine if any drift in the machine had occurred, followed by the daily calibration block. If results varied by more than 2%, the analysis was repeated.

Three-day physical activity records were collected from all subjects at baseline and 12 weeks. Briefly, participants were counseled to record activity in 15 minute time periods throughout the day utilizing an activity code defined by 9 categories [19]. The categories range from 1 = lying down, (1.1 kJ/kg/15 min) to 9 = intense work/activity (8.2 kJ/kg/15 min). All activity logs were reviewed at one site (Purdue) and returned for clarification as needed. Thus an estimate of 24 hour energy expenditure can be calculated based on the results. Participants were asked to maintain their current activity status, and report any changes. Estimated energy expenditure using this approach was 14,591 ± 3,312, 15328 ± 3,136 and 14,022 ± 2,69 kJ/d for the LC, HC and HD groups, respectively, and there were no significant changes in this parameter from week 0 to week 12 (p = 0.96). Blood pressure and heart rate measurements were taken after the patient had been seated in an upright position in a chair for at least five minutes with the arm supported at heart level. Blood pressure was measured with an appropriately sized cuff using a standard, calibrated sphygmomanometer (Welch Allyn) on the same arm for every measurement. Two readings, at least one minute apart were taken and the average value reported. If the two readings differed by more than 10 mm Hg (systolic or diastolic pressure), additional readings were taken until there were two successive determinations within 10 mm Hg of each other. 

Plasma insulin was measured via standard radioimmunoassay using a commercially available kit (Linco Research, Inc., St. Charles, MO, USA). Parathyroid hormone level was determined using a commercial immunoradiometric assay, and calcitonin via standard radioimmunoassay (Nichols Institute Diagnostics, San Juan Capistrano, CA, USA). 1,25-(OH)_2_-D was determined via radioimmunoassay. Fasting glucose and lipid profiles (cholesterol, LDL-cholesterol, HDL-cholesterol and triglyceride) were assessed by standard clinical techniques in the clinical laboratory. Circulating glycerol was measured using a fluorometric method, as previously described [1,3,4].

*Data Analysis***:** Prior to statistical analysis, subject adherence to the protocol was assessed based upon the following *a priori* criteria. For the low dairy group (low calcium or high calcium), compliance was defined as <600 mg calcium in the daily diet, <1 daily serving of dairy in the diet, energy intake within 837 kJ of energy prescription, and return pill counts reflecting utilization of 80-100% of the placebo or calcium supplements provided each week. For the high dairy group, compliance was defined as >900 mg calcium in the daily diet, ≥3 daily servings of dairy in the daily diet, energy intake within 837 kJ of energy prescription, and return pill counts reflecting utilization of 80-100% of the placebo supplements provided each week. Only subjects who met all compliance criteria for a given week were recorded as compliant for that week. Total study compliance was then defined as meeting weekly compliance for 75% of the weeks (i.e., 9 out of the 12). 

Data were analyzed for statistical significance for 1) subjects adhering to the protocol, as described above, 2) for all subjects completing the study, and 3) for all subjects enrolled in the study (intent-to-treat analysis). For intent-to-treat, the last value carried forward method was used to impute the week 12 value prior to analysis. For all three analyses, the change in each response variable (i.e., week 12 minus baseline) was fit to a two-way analysis of variance (ANOVA) model with treatment and study site factors. Several baseline measurements were also considered as potential covariates in this model. When treatment factors were found to be significant, Tukey’s multiple comparison procedure was used to determine statistically significant treatment group differences. As shown in Table 1, one of the sites (Ohio State) experienced a low enrollment and adherence rate. Results from this site could not be included in the analyses that included a potential site by treatment interaction due to the lack of subjects in some cells. As a result, this site was excluded from the compliant and completer analysis, but is included in all intent-to-treat analyses. 

## 3. Results

Of the 106 subjects enrolled in the study, 93 completed the trial and 68 met the criteria outlined for adherent subjects. 

**Figure 1:**
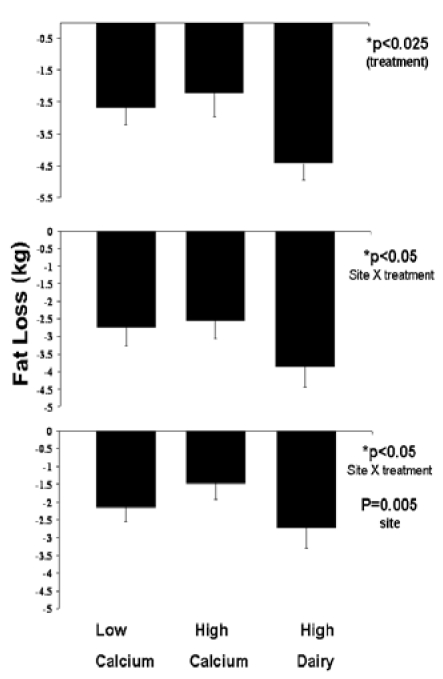
Effects of dietary treatments on fat loss. Top panel: Adherent subjects. Middle panel: All subjects completing the study. Bottom panel: Intent-to-treat analysis. All data are presented as mean ± standard error.

**Figure 2:**
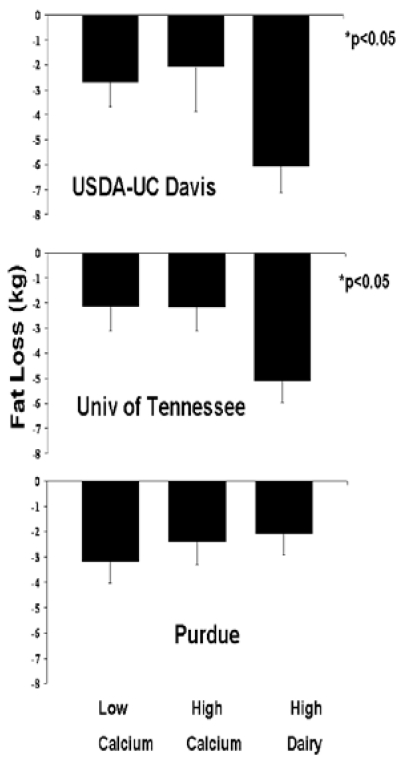
Site-Specific effects of dietary treatments on fat loss in adherent subjects. Top panel: USDA Davis. Middle panel: University of Tennessee. Bottom panel: Purdue. All data are presented as mean ± standard error.

*Fat Loss*: Among adherent subjects, the high dairy diet resulted in nearly a two-fold greater loss of fat mass compared to the low or high calcium diets, while the high calcium diet was without effect (HD: -4.43 ± 0.53 kg; LC: -2.69 ± 0.53 kg; HC: -2.23 ± 0.73 kg, p = 0.02 for overall treatment effect, p = 0.059 HD vs. LC, p = 0.047 HD vs. HC, p = 0.87 LC vs. HC, Figure 1). There was also a site by treatment interaction trend (p = 0.07) that was significant for completers (p = 0.03) and intent-to-treat (p = 0.04). This interaction in the adherent and completed analyses is explained by subjects on the high dairy diet at two sites (USDA Davis and University of Tennessee) exhibiting approximately two-fold increases in fat loss, while no such difference was observed at Purdue (Figure 2). The inclusion of Ohio State in the intent-to-treat also resulted in a significant site effect (p < 0.01) as subjects from that site did not exhibit significant fat loss regardless of treatment. 

**Figure 3:**
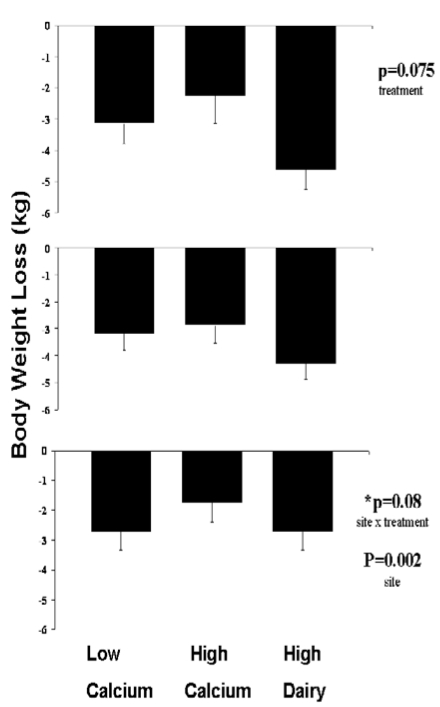
Effects of dietary treatments on weight loss. Top panel: Adherent subjects. Middle panel: All subjects completing the study. Bottom panel: Intent-to-treat analysis. All data are presented as mean ± standard error.

*Weight Loss*: There was a trend (p = 0.07 for treatment effect) toward augmented weight loss among adherent subjects on the high dairy (HD) diet (HD: -4.61 ± 0.63 kg; LC: -3.15 ± 0.62 kg; HC -2.27 ± 0.89 kg; baseline BMI utilized as a covariate in this analysis, Figure 3). This trend was not significant (p = 0.25) among those completing the study. The intent-to-treat analysis revealed a significant site effect (p < 0.01) and site by treatment interaction trend (p = 0.06). These results are again due to the inclusion of Ohio State as subjects in general did not lose weight. 

**Figure 4:**
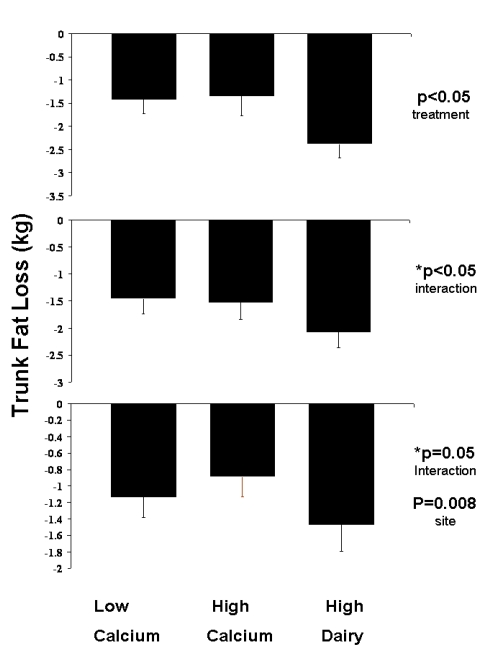
Effects of dietary treatments on trunk fat loss. Top panel: Adherent subjects. Middle panel: All subjects completing the study. Bottom panel: Intent-to-treat analysis. All data are presented as mean ± standard error.

*Trunk Fat Loss*: The results of trunk fat are similar to that of total fat. There was a significant treatment effect on fat loss from the central (trunk) region (p = 0.05 for overall treatment effect, p = 0.07 for HD vs. LC, p = 0.13 for HD vs. HC, p = 0.99 for LC vs. HC)) among those adhering to the study protocol with the high dairy diet resulting in a 1 kg greater trunk fat loss compared to the low calcium or high calcium groups (Figure 4). There was also a significant site by treatment interaction among those completing the study (p = 0.03), and an interaction trend in the intent-to-treat analysis (p = 0.05). Finally, a site effect was evident in the intent-to-treat analysis (p < 0.01) due to the inclusion of Ohio State. 

**Figure 5:**
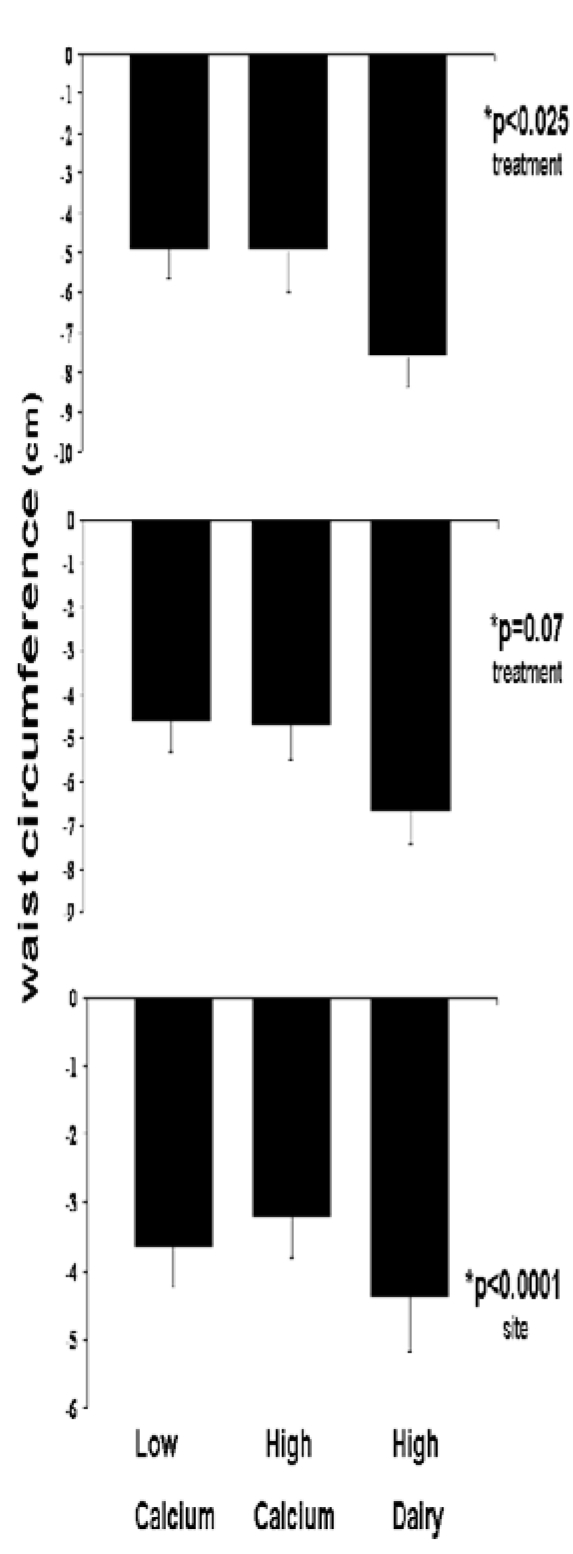
Effects of dietary treatments on change in waist circumference. Top panel: Adherent subjects. Middle panel: All subjects completing the study. Bottom panel: Intent-to-treat analysis. All data are presented as mean ± standard error.

*Waist Circumference Loss:* Among adherers, there was a significant decrease in waist circumference for subjects on the high dairy diet relative to the high and low calcium groups (p = 0.02 for overall treatment effect, p = 0.03 for HD vs. LC, p = 0.10 for HD vs. HC, p = 0.99 for LC vs. HC). The high dairy group, on average, lost of 3.5 cm compared to the other groups (Figure 5). This same trend was observed in the completers analysis (p = 0.07). In the intent-to-treat analysis, only a site effect was significant (p < 0.01). Subjects at Ohio State did not have a waist circumference reduction. 

*Lean Mass*: Although the high dairy group lost less lean mass (14 ± 345 g) than the high calcium (330 ± 476 g) or low calcium (527 ± 342 g) groups of adherent subjects, there were no statistically significant differences among treatment groups overall in the adherent, completer, or intent-to-treat analysis.

*Blood Pressure*: Systolic pressure is shown in Table 4 and diastolic pressure in Table 5. Although blood pressure decreased modestly with weight loss in this normotensive sample, as anticipated, there was an unanticipated response to the dietary treatments, in that the high calcium group exhibited significantly diminished blood pressure reductions in both the adherer and completer groups (Table 5).

**Table 4:** Changes in Systolic Blood Pressure (mmHg) in Response to Dietary Treatments^1^ Analysis.

	Adherers	Completers	Intent-to-Treat
**Low Calcium**	-6.23 ± 1.90	-5.43 ± 1.62	-0.04 ± 1.31
**High Calcium**	1.72 ± 2.54	-0.50 ± 1.75	-3.59 ± 1.28
**High Dairy**	-3.97 ± 1.88	-3.52 ± 1.61	-3.381 ± 173
**Significance**	p = 0.051(treatment)	p = 0.005(site)	p = 0.007(site)
	p = 0.027(site)	p = 0.048(treatment x site interaction)	p = 0.014(treatment x site interaction)

^1 ^Data presented as mean ± standard error

**Table 5:** Changes in Diastolic Blood Pressure (mmHg) in Response to Dietary Treatments^1 ^Analysis.

	Adherers	Completers	Intent-to-Treat
**Low Calcium**	-3.46 ± 1.51	-2.62 ± 1.31	-1.53 ± 1.05
**High Calcium**	2.01 ± 2.13	1.42 ± 1.49	1.25 ± 1.10
**High Dairy**	-3.49 ± 1.51	-3.13 ± 1.35	-2.71 ± 1.46
**Significance**	p = 0.082 (treatment)	p = 0.056	N.S.

**^1 ^**Data presented as mean ± standard error

*Biochemical Parameters*: Biochemical parameters in the adherent subjects are summarized in Table 6. Changes in circulating glycerol were used as an index of lipolysis. There was a significantly greater increase in glycerol in the adherent high dairy and high calcium groups compared to the low calcium group (p < 0.01). This effect was also evident among those completing the study (p < 0.0001) and in the intent-to-treat analysis (p < 0.0001). There were no significant treatment effects on circulating 25-OH-D, although levels were significantly lower (p < 0.0001) at Purdue than USDA Davis and The University of Tennessee (Purdue: 19.9 ± 0.8 ng/mL; USDA Davis: 26.6 ± 1.3 ng/mL; University of Tennessee: 24.5 ± 0.9 ng/mL). However, there were significant decreases in both PTH (p = 0.003) and1, 25-dihyroxyvitamin D (p < 0.0001) levels in response to both the high calcium and the high dairy diets. For both PTH and 1, 25-dihydroxyvitamin D, the high dairy diet resulted in ~two-fold greater decreases than found with the high calcium diet. PTH exhibited a site by treatment interaction trend (p < 0.10) and a significant site effect (p < 0.05); however, there was no consistent pattern of PTH across sites (site data not shown). 

**Table 6:** Change in Plasma Glycerol and Hormones in Response to Dietary Treatments^1,2,3^.

	Low Calcium	High Calcium	High Dairy	Significance
**Glycerol (µmol/L)**	-15 ± 8^a^	31 ± 10^b^	40 ± 8^b^	p < 0.0001
**25-OH-D (ng/mL)**	0.94 ± 0.81	1.62 ± 1.12	0.63 ± 0.81	NS
**1,25-(OH)_2_-D (pg/mL)**	0.46 ± 0.53^a^	-1.73 ± 0.73^b^	-4.01 ± 0.53^c^	p < 0.0001
**Insulin (**μ**U/mL)**	-1.48 ± 0.74	-1.47 ± 1.02	-1.99 ± 0.74	NS
**Leptin (ng/mL)**	-5.7 ± 2.5	-5.0 ± 3.5	-11.3 ± 2.5	NS
**Parathyroid Hormone (pg/mL)**	1.74 ± 1.85^a^	-3.80 ± 2.69^b^	-8.29 ± 1.97^c^	p < 0.005

^1^Adherent subjects ^2^Data presented as mean ± standard error. ^3^Non-matching superscript in each row denote significant differences.

**Figure 6:**
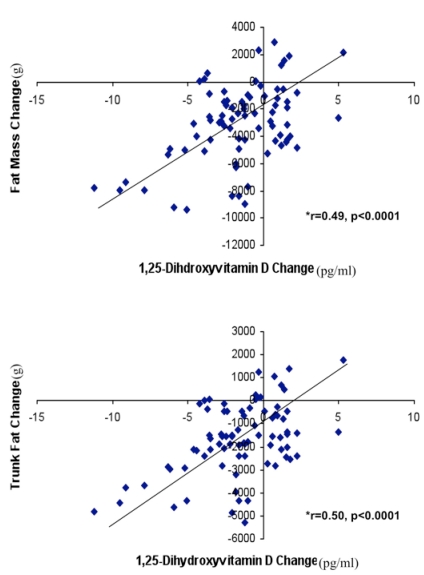
Correlation between change in plasma 1,25-dihydroxyvitamin D and change in total body fat (upper panel) and trunk fat (lower panel) among all subjects completing the study.

There was a significant correlation between the change in 1,25-dihydroxyvitamin D and the change in total body fat among those adhering to the study protocol (r = 0.58, p < 0.0001) as well as among all completers (r = 0.49, p < 0.0001) (Figure 6). When assessed by diet, there was a high degree of correlation between change in 1,25-dihydroxyvitamin D and the change in total body fat in subjects consuming the high dairy diet (r = 0.785, p < 0.0001 for adherent subjects; r = 0.710, p < 0.001 for completers) and a significant correlation for those consuming the high calcium diet (r = 0.671, p < 0.005 for adherent subjects; r = 0.401, p < 0.10 for completers). Similar relationships were found between the change in 1, 25-dihydroxyvitamin D and the change in trunk fat (r = 0.60 for adherent subjects, p < 0.0001; r = 0.50 for completers, p < 0.0001) (Figure 6).

Circulating insulin decreased among all treatment groups, consistent with the decreases in total body fat, and there was no significant difference among dietary treatment groups. Dietary treatment was without effect on circulating glucose, cholesterol, LDL-cholesterol, HDL-cholesterol and triglycerides (data not shown).

## 4. Discussion

Results of this study demonstrate that increasing the dairy content of the diet without altering dietary macronutrients significantly augments fat loss during energy restriction. This randomized, placebo-controlled trial has several strengths. The study utilized four centers and consequently included a substantially greater number of subjects than the previously published clinical trials (106 vs. 32-34) [2,3,4]. The diet-induced differences in body fat which occurred in this multi-center trial were sufficiently robust to support the conclusion of an effect of dairy on body fat mass not only in subjects who most closely adhered to the study protocol, but also in those who completed the study with a lower level of compliance and in an intent-to-treat analysis of all subjects initially enrolled. Although the body weight data show a similar trend, these data did not achieve statistical significance (0.05 < p < 0.10). This may be due to power considerations, as the original power calculations, based upon our previous clinical trials, assumed a 3.2 kg treatment difference. However, the low enrollment and adherence at one site resulted in a smaller than expected enrollment (Table 1), and the treatment differences were somewhat smaller than assumed (2.3 kg vs. 3.2 kg), resulting in a loss of power. 

Although the effects of dairy on adiposity in the present study are generally consistent with our previous findings, a notable difference is the lack of effect of supplementary calcium on weight or fat loss in the present study. We previously noted that both dairy-rich and calcium-supplemented diets augmented weight and fat loss secondary to energy restriction in both clinical trials and rodent studies [1,2,3,4,11,20], although dairy products exerted greater effects. Similarly, Davies *et al.* [9] and Heaney [10] noted protective effects of supplementary calcium against excess adiposity in their retrospective re-analysis of a series of nine calcium intervention studies originally designed with a skeletal endpoint. In contrast, data from the present study demonstrate an effect of dairy foods on body fat mass but failed to demonstrate any effect of supplementary calcium on adiposity. The reason for this discrepancy is not clear, although it should be noted that the present study was of shorter duration than previous clinical trials. Further, it is notable that the supplementary calcium group also exhibited diminished blood pressure responses to weight reduction compared to the other two groups. 

Lack of adiposity response to the calcium supplement may be related to incomplete suppression of 1,25-dihydroxyvitamin D. Our previous data suggest that 1,25-dihydroxyvitamin D promotes energy storage and adiposity by modulating adipocyte lipogenesis, lipolysis and apoptosis [1,11,22,23,24]; accordingly, we proposed that the anti-obesity effect of dietary calcium results in large part from suppression of 1,25-dihydroxyvitamin D [reviewed in 1], Consistent with this, Melanson *et al.* [25] demonstrated that switching subjects from a low dairy diet to a high Ca/high dairy diet under energy deficit conditions in a randomized crossover whole room calorimetry study resulted in a significant increase in fat oxidation which was associated with a significant decrease in circulating 1,25-dihydroxyvitamin D [25]. This concept is further supported by the significant correlations between the changes in 1,25-dihydroxyvitamin D and both total fat and trunk fat in the present study. We utilized comparable levels of dietary calcium in both the high calcium and high dairy arms; consequently, both treatment arms should have caused comparable suppression of 1,25-dihydroxyvitamin D. However, the data demonstrate significantly greater suppression with the high dairy diet compared to the high calcium diet. While the reason for this discrepancy is not clear, this suggests that the high calcium diet utilized in the present study failed to sufficiently suppress 1,25-dihydroxyvitamin D levels and, consequently, did not augment weight or fat loss. 

Dairy foods contain a number of compounds which may explain the greater effect of dairy foods compared to calcium on adiposity. In addition to the aforementioned clinical and observational studies, previous rodent studies also demonstrated markedly greater effects of dairy on attenuating adiposity compared to supplementary calcium [1]. This additional non-calcium-mediated bioactivity has not been definitively identified, although the high concentration of leucine and of angiotensin converting enzyme (ACE) inhibitors in dairy foods appear to contribute to the additional effect [1,26]. Conjugated linoleic acid (CLA) is another component of dairy which may modestly influence body weight and composition, but this is unlikely to be a relevant factor in the amplification of calcium’s effects on body weight and composition, as we have found this amplification with the use of fat-free dairy products which contain no CLA [11,20,21].

We found striking differences in response to the high dairy diet among the three sites that randomized sufficient subjects to all sites. Subjects on the high dairy diets at USDA Davis and The University of Tennessee exhibited ~two-fold increases in weight, fat and trunk fat loss loss, while those at Purdue did not. There are several possible explanations for this discrepancy. This study included both overweight and obese subjects, but randomization was not stratified for overweight status to ensure equal representation of overweight and obese subjects in each treatment group at each site. Although there were no significant differences in baseline body mass index in subjects assigned to the three diets at all sites combined (Table 2) or in body mass index across sites, application of the randomization schedule at Purdue resulted in only overweight (no obese) subjects in the high dairy group, while the other two treatment groups at Purdue and all groups at the other sites included obese subjects. Consequently, when this site is assessed separately from the aggregate study, the three treatment groups cannot be considered to be comparable at baseline, and it is possible that the lower weight and BMI of the high dairy group at Purdue versus the other sites may have contributed to an attenuation of total weight and fat loss in this group. However, this issue was addressed, in part, in the analysis in that baseline BMI was utilized as a covariate in the weight analysis. Additional factors that may have contributed to the differences between Purdue and the other sites may be the significantly younger age (22.7 vs. 26.3–27.9 years at the other sites) and significantly smaller initial body fat mass (29.8 vs. 32.2-35.0 kg) and waist circumference (84.5 vs. 91.0–94.0 cm) at Purdue vs. the other sites.

In addition, this study was conducted in the winter months, and of the three sites that randomized sufficient subjects to all dietary treatments, Purdue has the least opportunity for subjects to receive wintertime sun exposure. Indeed, although there were no effects of dietary treatments on circulating 25-hydroxyvitamin D levels, there were site differences, with Purdue exhibiting significantly lower levels than the other sites. Recent data indicate that such differences in vitamin D status may affect adiposity, as significant inverse relationships between 25-hydroxyvitmain D and total body fat and BMI have now been reported in several studies [27,28,29]. Accordingly, decreased vitamin D status may have contributed to the lack of responsiveness to the dairy intervention at the Purdue site. In support of this concept, the thermic effect of a meal was correlated to baseline 25-hydroxyvitamin D levels in the Purdue study cohort [30]. However, covarying with 25-hydroxyvitamin D levels in the model in the current study did not alter the results across sites, suggesting that other factors may have also contributed to this site difference.

Boon *et al.* [31] reported a significant relationship between calcium intake and body composition in the Amsterdam Growth and Health Longitudinal Study, a 23-year follow-up study of >2,000 individuals followed from age 13 to age 36. In contrast to the previously cited studies which have been conducted in populations with relatively low calcium intakes, the average calcium intake during the 23 year period was 1,269 mg for men and 1,148 mg for women. Nonetheless, the authors reported a modest but significant inverse relationship between calcium intake and body composition (sum of four skin-folds) for both men and women, with a significant negative interaction with age indicating a greater inverse relationship at older ages. However, there was no difference between the middle (800-1,200 mg calcium/day) and high (>1,200 mg/day) groups of calcium intake, suggesting that there is a calcium threshold of approximately 800 mg/day above which additional calcium confers diminishing returns which are difficult to detect, especially in smaller studies [31]. 

Bowen *et al.* [32] reported that dairy failed to enhance weight loss during 12 weeks of energy restriction in subjects on high protein (34 energy %) diets; the baseline calcium intakes were ~900 and 800 mg/day for men and women, respectively, suggesting that the additional dairy was supplementing marginally low intakes rather than correcting deficient intakes. Although calcium intake was successfully reduced to 500-600 mg/day in the low dairy protein group, the higher baseline calcium intakes may have blunted the opportunity to detect significant differences. However, another potential explanation for the lack of effect of additional dairy in this trial is the level of protein utilized. That study utilized a much higher level of protein intake than that used in the previous clinical trials (34% vs. 18% of energy), making a direct comparison difficult, as higher protein intakes have been shown in some studies to be associated with greater weight loss. Indeed, the weight loss found in that 12-week study was approximately twice as high (9.77 kg) as that found in the control group in our earlier 12-week study (4.99 kg) and three times as high as in the present multi-center trial (3.18 kg). At this higher rate of weight loss (0.8 kg/week), a maximal rate of fat mobilization may already be approached, making additional increments due to dairy (or other factors) unlikely.

In conclusion, results of this study demonstrate that increasing the intake of dairy foods while restricting dietary energy for weight loss results in augmentation of weight and fat loss in overweight and obese subjects. These multi-center results support earlier findings of small-scale clinical trials which show that dairy foods augment weight and fat loss during weight loss diets, but are discordant with previous findings regarding supplementary calcium. The reason for the latter discrepancy is not yet clear, but may be related to reduced suppression of 1,25-dihydroxyvitamin D levels by the high calcium versus high dairy diet. 
